# Body weight of newborn and suckling piglets affects their intestinal gene expression

**DOI:** 10.1093/jas/skac161

**Published:** 2022-05-03

**Authors:** Sandra Villagómez-Estrada, José F Pérez, Diego Melo-Durán, Francesc Gonzalez-Solè, Matilde D’Angelo, Francisco J Pérez-Cano, David Solà-Oriol

**Affiliations:** Department of Animal and Food Science, Animal Nutrition and Welfare Service (SNIBA), Autonomous University of Barcelona, Bellaterra 08193, Spain; Carrera de Medicina Veterinaria, Escuela Superior Politécnica de Chimborazo (ESPOCH), Riobamba 060155, Ecuador; Department of Animal and Food Science, Animal Nutrition and Welfare Service (SNIBA), Autonomous University of Barcelona, Bellaterra 08193, Spain; Department of Animal and Food Science, Animal Nutrition and Welfare Service (SNIBA), Autonomous University of Barcelona, Bellaterra 08193, Spain; Faculty of Medical Sciences “Eugenio Espejo,” UTE University, Quito 17012764, Ecuador; Department of Animal and Food Science, Animal Nutrition and Welfare Service (SNIBA), Autonomous University of Barcelona, Bellaterra 08193, Spain; Department of Animal and Food Science, Animal Nutrition and Welfare Service (SNIBA), Autonomous University of Barcelona, Bellaterra 08193, Spain; Department of Biochemistry and Physiology, University of Barcelona, Barcelona 08007, Spain; Department of Animal and Food Science, Animal Nutrition and Welfare Service (SNIBA), Autonomous University of Barcelona, Bellaterra 08193, Spain

**Keywords:** birthweight, gene expression, hyperprolific sows, neonatal pigs, suckling pigs

## Abstract

Modern hyperprolific sows must deal with large litters (16–20 piglets) which reduce piglet birthweight with a concomitant increase in the proportion of small and intrauterine growth retarded piglets. However, larger litters do not only have a greater variation of piglet weights, but also a greater variation in colostrum and milk consumption within the litter. To further understand the impact that body weight has on piglets, the present study aimed to evaluate the degree of physiological weakness of the smallest piglets at birth and during the suckling period (20 d) compared to their middle-weight littermates through their jejunal gene expression. At birth, light piglets showed a downregulation of genes related to immune response (*FAXDC2*, *HSPB1*, *PPARGC1α*), antioxidant enzymes (*SOD2m*), digestive enzymes (*ANPEP, IDO1, SI*), and nutrient transporter (*SLC39A4*) (*P* < 0.05) but also a tendency for a higher mRNA expression of *GBP1* (inflammatory regulator) and *HSD11β1* (stress hormone) genes compared to their heavier littermates (*P* < 0.10). Excluding *HSD11β1* gene, all these intestinal gene expression differences initially observed at birth between light and middle-weight piglets were stabilized at the end of the suckling period, when others appeared. Genes involved in barrier function (*CLDN1*), pro-inflammatory response (*CXCL2, IL6, IDO1*), and stress hormone signaling (*HSD11β1*) over-expressed compared to their middle-weight littermates (*P* < 0.05). In conclusion, at birth and at the end of suckling period, light body weight piglets seem to have a compromised gene expression and therefore impaired nutrient absorption, immune and stress responses compared to their heavier littermates.

## Teaser

At birth and during lactation phase, the light body weight of piglets born from hyperprolific sows predisposed to a higher expression of stress and pro-inflammatory genes.

## Introduction

Intensive pressure for a genetic selection of hyper prolificity, based on sow reproductive performance (litter size; 16–20 total piglets born), has led to a reduction in the average piglet birthweight as well as an increase in the variability of birthweights within the litter (Peltoniemi et al., 2021). Thus, the proportion of small piglets in large litters as well as the frequency of intrauterine growth-restricted (IUGR) piglets has notably increased (Matheson et al., 2018; Ward et al., 2020). The initial birthweight variation within litter may reveal that in hyperprolific genetic lines, sows may not be able to ensure a satisfactory nutrition for the uneven litter, resulting in an intense pre- and post-natal sibling competition to acquire limited nutrients and a high mortality risk for weaker offspring (Ward et al., 2020). Additionally, most of these IUGR and light birthweight piglets may also show long-term negative effects on their organ structure, postnatal growth, and feed efficiency (Ji et al., 2017). Thus, modern commercial farms need to cull IUGR piglets because there is no effective nutritional or management support for their growth or survival during the suckling and post-weaning periods (Kraeling and Webel, 2015). Nonetheless, light birthweight piglets could show compensatory growth if given optimal management and dietary conditions (van Barneveld and Hewitt, 2016; Viott et al., 2018; Farmer and Edwards, 2021). Fetal or neonatal programming occurs during the gestation period leading to changes in gene transcription resulting in altered activities of metabolic pathways and homeostatic control processes (Burdge et al., 2007). Insufficient maternal nutrition during gestation can result in permanent fetal programming alterations (Kwon and Kim, 2017). In order to further understand the impact that the intrauterine fetal overcrowding has on piglets, the present study aimed to evaluate the changes in jejunal gene expression, as a key indicator of the intestinal function and development. Therefore, the gene expression of light-weight piglets at birth and at the end of the lactation period was compared with that of their middle-weight littermates, both born to hyperprolific sows under commercial conditions.

## Materials and Methods

All animal experimentation procedures were approved by the Ethics Committee of the Universitat Autònoma de Barcelona (CEEAH2788M2) in compliance with the European Union guidelines for the care and use of animals in research (European Parliament, 2010).

### Animals and housing

At 113 d post-mating (± 1.27), the litter characteristics of 80 litters (1,542 total born neonates) from 80 hyperprolific sows (DanBred hybrid line; Landrace × Yorkshire, parity 4.78 ± 1.69) were recorded. Sows and their litters were followed until weaning (21.6 ± 1.27 d). During the gestation and lactation periods, sows were kept under commercial feeding and management conditions. Gestation feed intake was individually controlled using a feeding electronic station (model Intec-Mac, Mannebeck, Schuttorf, Germany), whereas during the lactation period, the feeders were manually filled twice a day (8:00 and 15:00 h) to guarantee ad libitum intake. Sows were fed 2.4 kg/d from weaning to 35 d post-mating and 2.1 kg/d from 35 to 110 d post-mating. Litter performance characteristics were recorded at farrowing and weaning. Within 48 h after birth, piglets were processed (e.g., iron administration, ear tagged) and cross-fostered to standardize litter size to 15 piglets/litter (in average, functional teats + 1 piglet as standard handling and management of the farm). Water was provided ad libitum through a commercial nipples waterer.

### Experimental diet and measurements

Sow BW was recorded at 35, 110 d of gestation and at weaning. Individual feed intake was recorded daily according to the electronic feeding station during the gestation phase (35 to 110 d of gestation) while during lactation feed intake was recorded manually by daily weighting of the difference between the feed offered and its disappearance. Diets were formulated to cover nutrient requirements (NRC, 2012). Gestation diet main composition includes wheat (35%), barley (23.5%), wheat bran (20%), sunflower cake (10%), maize (7.70%), and lysine 50 (0.44%). The rest of ingredients, amino acids, and macro and micro minerals accounted for 3.36% of the total composition. The nutrient content of gestation diet was 2,260 kcal/kg net energy, 13.0% crude protein, and 0.67% lysine. Regarding lactation diet, the main ingredients were wheat (37%), maize (30%), soybean meal (11.5%), sunflower cake (7%), lysine 50 (0.92%), and other ingredients (13.58%). Lactation diet values for net energy were 2,450 kcal/kg, 15.5% for crude protein, and 1.08% for lysine.

### Sampling

From the total of 80 initially housed sows, a subset of 10 multiparous sows (3rd to 5th parity) were chosen for sampling purposes. Thus, two littermates from each litter were selected to take jejunum samples during farrowing (before colostrum intake) and during suckling period (20 d). The selection criterion was the piglet body weight (BW) categorized into two levels: light and middle-weight littermates. Briefly, a light piglet was defined as having a birth weight between 600 and 800 g (belonging to the lower quartile) at birth and a BW between 2,500 and 3,800 g on day 20, whereas a middle-weight littermate had a BW within the average of the litter at birth (1,200–1,300 g) and at the end of lactation (4,000–5,100 g). Piglets with no obvious characteristics of disease or injury were selected. Piglet sex was not considered as sampling indicator. Individual BW was examined and if it matched into the light or middle BW category, piglet was selected to obtain samples.

Selected piglets were removed from the sow, approximately 150 min (±15) after the start of farrowing, and euthanized by an overdose of sodium pentobarbital (Dolethal, Vetoquinol, S.A., Madrid, Spain). Samples for gene expression analysis were collected as described in Villagómez-Estrada et al., (2021). Briefly, approximately at the midpoint of the jejunum, one sample of approximately 1.5 cm was collected, rinsed in PBS solution, snap frozen in 1 mL of RNA later (Deltalab, Barcelona, Spain), and stored at −80 °C until processing.

### Gene expression analysis

Gene expression in jejunal tissue was performed on 56 genes using an Open Array Real-Time PRC Platform (Applied Biosystems, Waltham, MA). All genes were involved in multiple physiological functions closely related to intestinal health and were selected based on the literature and grouped according to their main function as follows: 1) barrier function genes such as the family members of claudins (*CLDN*), mucins (*MUC*), zonula occludens (*ZO*), trefoil factor (*TFF*), and occludin (*OCLN*) (*CLDN1*, *CLDN4*, *CLDN15*, *MUC2*, *MUC13, ZO1*, *TFF3,* and *OCLN*); 2) genes involved in immune and inflammatory responses such as pattern recognition receptors, cytokines, chemokines, and stress proteins [toll-like receptor (*TLR2*, *TLR4*); interleukin (*IL-1β*, *IL6*, *IL8*, *IL10*, *IL17A*, *IL22*); interferon gamma (*IFNG, IFNGR1*); tumor necrosis factor (*TNF*); transforming growth factor beta 1 (*TGF-β1*); chemokine ligand (*CCL20, CXCL2*); heat shock protein (*HSPB1*, *HSPA4*); regenerating family member 3 gamma (*REG3G*); peroxisome proliferator activated receptor gamma (*PPARGC1α*); fatty acid hydroxylase domain containing (*FAXDC2*) and guanylate binding protein (*GBP1*)]; 3) antioxidant enzymes genes (glutathione peroxidase, *GPX2*; superoxide dismutase, *SOD2*); 4) digestive enzymes and hormones genes involved in the digestion and metabolism processes [alkaline phosphatase intestinal (*ALPI);* sucrase-isomaltase (*SI*); d-amino-acid oxidase (*DAO1*); histamine N-methyltransferase (*HNMT*); alanyl aminopeptidase membrane (*ANPEP*); indoleamine 2,3-dioxygenase (*IDO1*); glucagon (*GCG*); cholecystokinin (*CCK*); insulin-like growth factor (*IGF1R*); and peptide YY (*PYY*)]; 5) nutrient transport coding genes [(solute carrier family (*SLC5A1*, *SLC16A1*, *SLC7A8*, *SLC15A1*, *SLC13A1*, *SLC11A2*, *SLC30A1*, *SLC39A4)* and Metallothionein (*MT1A*)]; and 6) stress response genes [corticotropin releasing hormone receptor (*CRHR1)*; nuclear receptor (*NR3C1*); and hydroxysteroid (11-beta) dehydrogenase (*HSD11β1*)]. Four housekeeping genes were used to calculate the relative values for gene data [actin beta (*ACTB*); beta-2-microglobulin (*B2M*); glyceraldehyde-3-phosphate dehydrogenase (*GAPDH*); and TATA-box binding protein (*TBP*)]. The RNA gene expression analysis was performed according to the methodology described in Villagómez-Estrada et al., (2021). Briefly, from 50 mg of frozen jejunum tissue, the RNA was obtained using the RiboPure kit (Ambion, Austin, TX) and following the manufacturer’s instruction. The quality and quantity of RNA was assessed with a NanoDropND-1000 spectrophotometer (NanoDrop products, Wilmington, DE), whereas the RNA integrity was checked with Agilent Bioanalyzer-2100 equipment (Agilent Technologies, Santa Clara, CA). Reverse transcription of approximately 1 µg of total RNA to single-stranded cDNA in a final volume of 20 μL was performed using a High-Capacity cDNA Reverse Transcription kit (Applied Biosystems, Foster City, CA) and random hexamer primers. The thermal cycler conditions applied were as follows: 25 °C 10 min; 37 °C 120 min; 85 °C 5 min; and 4 °C hold. Description of primers used are shown in Table 1. Gene expression analysis was performed in duplicates for each sample. Data were collected and analyzed using the ThermoFisher Cloud software 1.0 (Applied Biosystems) applying the 2^−ΔΔCt^ method for relative quantification and using the sample with the lowest expression as a calibrator. The maximum cycle relative threshold was adjusted at 26, amplification score < 1.240, quantification cycle confidence > 0.8, and the maximum standard deviation between duplicates was set at < 0.38.

**Table 1.** List of primers used in gene expression analysis by Open Array Real-Time PCR custom designed

Gene	Name	Primer Forward (5´-3´)	Primer Reverse (5´-3´)	Probe (5´-3´)
*CLDN1*	Claudin-1	CTTCGACTCCTTGCTGAATCTGA	CTTCCATGCACTTCATACACTTCAT	ACAGCACTTTGCAAGC
*CLDN4*	Claudin-4	CCTCCGTGCTGTTCCTCAA	GAGGCACAAGCCCAGCAA	CCTTGTGGCACTTTG
*CLDN15*	Claudin-15	GCTATCTCCTGGTATGCCTTCAA	GGGACTTCCACACTCCTTGGT	ACTTCTTCGACCCCTTGTA
*MUC2*	Mucin 2	AAGGACGACACCATCTACCTCACT	GGCCAGCTCGGGAATAGAC	CATGGTCAGCACCCCG
*MUC13*	Mucin 13	CAGTGGAGTTGGCTGTGAAAAC	ATCAAGTTCTGTTCTTCCACATTCTTG	TCCTCTCATTAAGATCAAAC
*ZO1*	Zonula occludens 1	GCTATGTCCAGAATCTCGGAAAA	TGCTTCTTTCAATGCTCCATACC	TCACCATCTTTTTACAACTAC
*TFF3*	Trefoil factor 3	AGAACCTGCCCGTGACCAT	CACACTGGTTCGCCGACAG	AGGCCAGGATGTTCT
*OCLN*	Occludin	CAGGTGCACCCTCCAGATTG	CAGGCCTATAAGGAGGTGGACTT	TGACATCAGCCATGTCAT
*TLR2*	Toll-like receptor 2	CTCTCGTTGCGGCTTCCA	AAGACCCATGCTGTCCACAAA	CAAGGTCAACTCTCTG
*TLR4*	Toll-like receptor 4	CATCCCCACATCAGTCAAGATACT	TCAATTGTCTGAATTTCACATCTGG	ACAGCAATAGCTTCTCCA
*IL1B*	Interleukin 1 beta	GGTGACAACAATAATGACCTGTTATTTG	GCTCCCATTTCTCAGAGAACCA	ATGAAGTGCTGCACCC
*IL6*	Interleukin 6	CCAATCTGGGTTCAATCAGGAG	ACAGCCTCGACATTTCCCTTATT	AGATATACCTGGACTACCTC
*IL8*	Interleukin 8	GGAAAAGTGGGTGCAGAAGGT	GAGAATGGGTTTTTGCTTGTTGT	TACAGATATTTTTGAAGAGAACT
*IL10*	Interleukin 10	TGAGGCTGCGGCGCT	GAGCTTGCTAAAGGCACTCTTCA	AACAAGAGCAAGGCCGT
*IL17A*	Interleukin 17	CCAGACGGCCCTCAGATTAC	ATCTTCCTTCCCTTCAGCATTG	CCATGGACTCTCCAACG
*IL22*	Interleukin 22	TGTTCCCCAACTCTGATAGATTCC	GTTGTTCACATTTCTCTGGATATGCT	AGCTAAGCCAATGCCGTAT
*IFNG*	Interferon gamma	TGACTTTGTGTTTTTCTGGCTCTT	CACTCTCCTCTTTCCAATTCTTCAA	ATCCTAAAGGACTATTTTAAT
*IFNGR1*	Interferon gamma receptor 1	CATGTTACCCAAATCTTTGCTGTCT	CAGTATGCACGCTTGAAATTGTC	ATATATATCACCCATCACCTACC
*TNF*	Tumor necrosis factor alpha	CACCACGCTCTTCTGCCTACT	GACGGGCTTATCTGAGGTTTGA	CAAGGACTCAGATCATCGT
*TGFB1*	Transforming growth factor beta 1	GCGGCAGCTCTACATTGACTT	GACCTTGCTGTACTGAGTGTCTAGG	CCATGCCAATTTCTGCCT
*CCL20*	Chemokine (C-C motif) ligand 20	GACCATATTCTTCACCCCAGATTT	CACACACGGCTAACTTTTTCTTTG	ATCAATGCAATCATCTTT
*CXCL2*	Chemokine (C-X-C motif) ligand 2	CATGGTGAAGAAAATCATCGAGAA	GCCAGTAAGTTTCCTCCATCTCTCT	AACAAGAGCAGTGCCAAC
*HSPB1*	Heat shock protein 27	CGAGGAGCTGACGGTCAAG	GCAGCGTGTATTTTCGAGTGAA	ACGGCTTCATTTCCCGGT
*HSPA4*	Heat shock protein 70	TCAATTGCCTGCGATTAATGAA	GAATGCCCCATGTCTACAAAAAC	CAGTTGCTCTTGCATATG
*REG3G*	Regenerating-islet derived protein 3 gamma	TGCCTGATGCTCCTGTCTCA	GGCATAGCAGTAGGAAGCATAGG	CCAAGGTGAAGATTC
*PPARGC1A*	Peroxisome proliferative activated receptor gamma, coactivator 1 alpha	CTCTGGAACTGCAGGCCTAA	TGGAGAAGCCCTAAAAGGGTTAT	ACCCACAACTCCTCCT
*FAXDC2*	Fatty acid hydrolase domain containing 2	CCATGACTACCACCATCTCAAGTT	GCAGGATCGTGTGTCTCTCGTA	TGTTCAAGCAGACCAAG
*GBP1*	Guanylate binding protein 1	AGAATCCATCACAGCAGACGAGTA	CGGATACAGAGTCGAGGCAGGTTAA	TCAAGCTTAAGAAGGGTACCAG
*GPX2*	Glutathione peroxidase 2	CAACCAATTTGGACATCAGGAG	GGGTAAAGGTGGGCTGGAAT	AGATCCTGAACAGCCTCA
*SOD2*	Superoxide dismutase	GGGTTGGCTCGGTTTCAA	CATGCTCCCACACGTCGAT	CTGCAAGGAACAACAGGTCT
*ALPI*	Intestinal alkaline phosphatase	ATGTCTTCTCTTTTGGTGGCTACA	GGAGGTATATGGCTTGAGATCCA	AAGCTCCGTTTTTGGCCT
*SI*	Sucrase-isomaltase	CGACCCCTTTTGCATGAGTT	AAGGCTGGACCCCATAGGAA	TTTAATGAAAAGCCAACCTG
*DAO1*	Diamine oxidase	GGAACCAACAGACCTTCAACTATCTC	TTCGGAATCCCAGGACCAT	CCGGACCCTTACTGGAAA
*HNMT*	Histamine N-methyltransferase	TGTTGAACCAAGTGCTGAACAAAT	ACTTTATGTTCTCGAGGTTTGATGTCTT	ACCAAGTACAAAGAGCTT
*ANPEP*	Aminopeptidase-N	AGGGCAACGTCAAAAAGGTG	GTCAAAGCATGGGAAGGATTTC	ACACAGATGCAGTCTACAG
*IDO1*	Indoleamine 2,3 dioxygenase	TTGGCAAATTGGAAGAAAAAGG	CCGGAAATGAGAAGAGAATATCCAT	CCAGTGGGCCCATGACTTAC
*GCG*	Glucagon	AGGCGTGCCCAGGATTTT	CATCGTGACGTTTGGCAATG	CACCAAGAGGAACAAGAA
*CCK*	Cholecystokinin	CAGCAGGCTCGAAAAGCAC	AATCCATCCAGCCCATGTAGTC	CAGCCACAGAATAAGTGA
*IGF1R*	Insulin-like growth factor 1 receptor	CCGACGCGGCAACAAC	TCAGGAAGGACAAGGAGACCAA	CTACGTGAAGATCCGCCA
*PYY*	Peptide tyrosine tyrosine	CAGAGGTATGGGAAACGTGACA	CCTTCTGGCCACGACTTGAC	CAAACTGCTCTTCCCTGAA
*SLC5A1*	Solute carrier family 5 (sodium/glucose cotransporter) member 1	GGCCATCTTTCTCTTACTGGCA	TCCCACTTCATGAAAAGCAAAC	TTTATACGGATACCTTGCAGAC
*SLC16A1*	Monocarboxylate transporter 1	CCTTGTTGGACCTCAGAGATTCTC	CCAGTATGTGTATTTATAGTCTCCGTATATGTC	CCACCACTTTTAGGTCGTC
*SLC7A8*	Solute carrier family 7 (amino acid transporter light chain, L System) member 8	TGTCGCTTATGTCACTGCAATGT	GACAGGGCGACGGAAATG	CTGTGACTTTTGGAGAGAA
*SLC15A1*	Solute carrier family 15 (oligopeptide transporter) member 1	GGTTATCCCTTGAGCATCTTCTTC	AGTGCTCTCATTCCATAGTAGGAAAA	TCAACGAGTTCTGTGAAAG
*SLC13A1*	Solute carrier family 13 (sodium/sulfate symporters) member 1	GGTACCTCCACCAACTTGATCTTC	ATCCAAAGTTGATGCAGTGACAAT	ATTTCAATATGCGCTACCC
*SLC11A2*	Solute carrier family 11 (proton-coupled divalent metal ion transporter) member 2	GTCTTTGCCGAAGCGTTTTTT	ACCACGCCCCCTTTGTAGA	CCAACCAGCAGGTGGT
*SLC30A1*	Solute carrier family 30 (zinc transporter) member 1	AATTGGACCGGACAGATCCA	TCTCTGATAAGATTCCCATTCACTTG	AAAAGTCCAGAAGTGATGC
*SLC39A4*	Solute carrier family 39 (zinc transporter) member 4	ATCTTTGGGCTCTTGCTCCTT	GCAGCCCCAGCACCTTAG	CTGCTACCCACTACGTCA
*MT1A*	Metallothionein 1A	TGAATCCGCGTTGCTCTCT	CAGGAGCAGCAGCTCTTCTT	ACGTGCAAAACCTGCAGA
*CRHR1*	Corticotropin releasing hormone receptor 1	CAGGGCCCCATGATATTGG	CCGGAGTTTGGTCATGAGGAT	CTGATCAACTTTATCTTCC
*NR3C1*	Glucocorticoid receptor	GGCAATACCAGGATTCAGGAACT	CCATGAGAAACATCCATGAATACTG	TGACCAAATGACCCTCCT
*HSD11B1*	Hydroxysteroid (11-beta) dehydrogenase 1	GGTCAGAAGAAACTCTCAAGAAGGTG	GCGAAGGTCATGTCCTCCAT	TCTTCAGCACACTACGTTG
*B2M*	Beta-2-microglobulin	TCACTCCTAACGCTGTGGATCA	CGGTTAGTGGTCTCGATCCC	AGCACGTGACTCTCGATA
*GAPDH*	Glyceraldehyde-phosphate-dehydrogenase	TTCGTCAAGCTCATTTCCTGGTA	TCCTCGCGTGCTCTTGCT	AATTTGGCTACAGCAACAG
*TBP*	TATA-box binding protein	CAGAATGATCAAACCGAGAATTGT	CTGCTCTGACTTTAGCACCTGTTAA	TTTGTCTCTGGAAAAGTTGT

### Statistical analysis

Data of gene expression were analyzed with one-way ANOVA considering BW piglet category as a main factor. The effect of sow was not considered within the statistical model. Two separate analyses were performed, at birth and at the end of the lactation period. Gene expression data were subjected to a logarithmic transformation to get closer to the Gaussian distribution. The Benjamini and Hochberg false discovery rate (FDR) multiple testing correction was also performed using the p.adjust function of R software v.3.4.3 (Benjamini and Hochberg, 1995). All statistical analyses were performed using R software and Bioconductor software (Gentleman et al., 2004). Significance was declared at a probability *P* ≤ 0.05, and tendencies were considered when *P*-value was between >0.05 and <0.10 using Tukey adjust.

## Results

Description of the sow and litter performance is shown in Table 2. The average BW of the sampled light and middle-weigh piglets at birth was 741 and 1,367 g, whereas at the end of suckling period was 3,125 and 4,733 g, respectively. All samples showed an adequate amplification. The effect of piglet BW on the intestinal expression of some genes involved in multiple physiological functions is shown in Table 3. At birth, light piglets showed a downregulation of genes from immune response (*FAXDC2*, *HSPB1*, *PPARGC1α*), antioxidant enzymes (*SOD2m*), digestive enzymes (*ANPEP, IDO1, SI*), and nutrient transporter (*SLC39A4*) (*P* < 0.05), but also a tendency for a higher mRNA expression of *GBP1* (immune/inflammation response) and *HSD11β1* (stress enzyme) compared to their heavier littermates (*P* < 0.10). In fact, the tendency for *HSD11β1* higher gene expression in light piglets was also observed at the end of the suckling period. At that age, the light piglets of the litter had a higher mRNA expression of five genes involved in barrier function (*CLDN1*), pro-inflammatory response (*CXCL2, IL6*), digestive enzyme (*IDO1*), and stress hormone signaling (*HSD11β1*) compared to the middle-weight piglets (*P* < 0.05). Likewise, a tendency for an up-regulation of *CRHR1* gene in light-weight piglets was observed (*P* < 0.10; Table 3).

**Table 2.** Reproductive performance and growth of sow and piglets during the studied period

Item	Mean	SD
Sows, *n*	80	–
Sow BW, kg		
Gestation (d 35)	248.3	36.97
Gestation (d 110)	270.9	28.80
Weaning (d 24)	259.7	33.32
Average daily feed intake gestation, kg	2.33	0.09
Average daily feed intake lactation, kg	7.45	1.22
Reproductive parameters		
At birth		
Total born pigs^1^, *n*	19.3	4.02
Pigs born alive, *n*	16.1	3.44
Born alive litter birth weight, kg	21.0	3.90
Born alive pig weight, kg	1.3	0.20
At weaning		
Total weaned pigs, *n*	14.2	1.69
Litter weaning weight, kg	74.7	14.63
Weaned pig mean BW, kg	5.3	0.82
BW Q1	3.6	0.52
BW Q2	4.8	0.27
BW Q3	5.7	0.23
BW Q4	7.0	0.73
At sampling^2^		
Litter, *n*	10	–
Birthweight light, kg	0.7	0.10
Birthweight middle, kg	1.3	0.17
Weaning light, kg	3.1	0.48
Weaning middle, kg	4.7	0.34

SD, standard deviation.

Total born includes born alive and stillborn piglets.

Data are arithmetic means of 10 piglets for each category and period.

**Table 3.** Relative gene expression in light and middle weight piglets at birth and at weaning (20 d)^1^

Function		Birth weight					Weaning weight				
	Gene	Light	Middle	SEM	*P*	FDR	Light	Middle	SEM	*P*	FDR
Barrier function											
Claudin-1	*CLDN1*	1.10	0.94	0.071	0.322	0.650	2.41	1.02	0.091	0.046	0.415
Immune response											
Fatty acid hydrolase domain containing 2	*FAXDC2*	1.70	3.12	0.063	0.010	0.138	107.57	129.83	0.152	0.702	0.887
Guanylate binding protein 1	*GBP1*	0.31	0.06	0.141	0.062	0.319	1.53	1.19	0.093	0.806	0.907
Heat shock protein 1	*HSPB1*	1.27	1.81	0.029	0.002	0.083	3.47	3.33	0.045	0.970	0.992
Peroxisome proliferative activated receptor gamma, coactivator 1 alpha	*PPARGC1α*	2.87	4.79	0.062	0.012	0.138	2.73	2.67	0.102	0.391	0.815
Interleukin 6	*IL6*	1.18	1.44	0.119	0.517	0.820	6.38	1.90	0.097	0.007	0.165
Chemokine ligand 2	*CXCL2*	1.20	0.90	0.078	0.263	0.650	10.99	4.08	0.101	0.025	0.283
Antioxidant enzyme											
Superoxide dismutase	*SOD2m*	1.17	1.37	0.027	0.100	0.462	1.40	1.20	0.059	0.673	0.887
Digestive enzyme											
Aminopeptidase-N	*ANPEP*	0.61	0.72	0.027	0.047	0.311	1.68	1.44	0.092	0.615	0.887
Indoleamine 2,3-dioxygenase	*IDO1*	1.40	4.72	0.120	0.040	0.310	12.90	3.30	0.115	0.024	0.283
Sucrase-isomaltase	*SI*	7.27	55.70	0.171	0.006	0.131	130.06	84.69	0.573	0.206	0.815
Nutrient transporter											
Solute carrier family 39 member 4 (Zn transporter)	*SLC39A4*	1.31	2.19	0.074	0.028	0.258	1.88	1.64	0.095	0.752	0.887
Stress enzyme											
Hydroxysteroid (11-beta) dehydrogenase 1	*HSD11β1*	2.88	1.92	0.029	0.062	0.319	3.60	1.73	0.056	0.002	0.079
Stress hormone											
Corticotropin releasing hormone receptor 1	*CRHR1*	2.33	2.39	0.094	0.982	0.985	7.15	3.63	0.120	0.060	0.451

Data are means of 10 piglets for each category and correspond to back-transformed values. Gene expression values are indicated as ratios of cycle relative threshold value for each gene normalized to that of the reference sample. Only significant (*P*-value < 0.05) and tendency (*P*-value < 0.10) differences are presented.

SEM, standard error of the mean; FDR, false discovery rate.

## Discussion

The intestinal mucosa possesses a complex function; it does not only play an important role in epithelial barrier and nutrient digestion but is also part of a well-organized immune system (Okumura and Takeda, 2017). Like BW, the development and functionality of the gastrointestinal tract can be impaired due to prenatal events (Dong et al., 2014; Farmer and Edwards, 2021). This becomes especially important at weaning when stress and pro-inflammatory events occurred. In the present study, intestinal gene expression at birth showed the immaturity of the light piglets intestine due to a reduced activation of digestive (*ANPEP*, *IDO1*, *SI*), nutrient transport (*SLC39A4*), immunity/inflammation (*FAXDC2*, *GBP1*, *HSPB1*, *PPARGC1α*), and antioxidant (*SOD2m*) genes accompanied also by an overexpression of the stress enzyme gene *HSD11β1*. For instance, the down-regulated digestive genes such as *ANPEP*, *IDO1*, and *SI* are mainly involved in the final digestive process of proteins, amino acids, and carbohydrates but may also act in an anti-inflammatory way (Trevisi et al., 2012; Xu et al., 2015). Moreover, the downregulation of the nutrient transport gene (*SLC39A4*) of Zn, considered as the major intracellular Zn transporter (Martin et al., 2013), may be involved in the changes observed in other physiological genes that need Zn for their proper expression (Suttle, 2010). Considering that the small intestine plays an essential role not only in diet digestion and nutrient absorption but also in immune response, this reduced gene expression observed in piglets of light birthweight may seriously compromise immunity functionality, as previously noted (Michiels et al., 2013; Qi et al., 2019; Li et al., 2021). Indeed, a decreased development of gastrointestinal tract (e.g., length, weight, and secretory capacity), even after 18 to 28 d post-weaning, has been observed in piglets with light birthweight compared to their average birth weight littermates (912 g vs. 1,287 g BW; Michiels et al., 2013). Noteworthy, an over-expression of *HSD11β1* gene was found in piglets with lower BW both at birth and at weaning. The *HSD11β1* gene is encoding for the enzyme that converts inactive cortisone to the active cortisol, therefore regulating tissue glucocorticoid levels and playing a critical role in metabolism and inflammatory response (Nixon et al., 2012; Huang et al., 2020). The fact that it remains elevated until the end of lactation shows that the fetal distress and the neonatal inflammatory condition are not reverted with age, hence suggesting that this negative impact may last until later in life. Since newborn piglets were not allowed to consume colostrum, further studies are needed to assess the influence of colostrum, the primary source of nutrients and immunoglobulins to piglets, in improving these physiological functions of piglets impaired by uterine overcrowding.

Excluding *HSD11β1* gene, all these intestinal gene expression differences initially observed at birth between light and middle-weight piglets disappeared at the end of the suckling period, indicating the normal acquisition of these functions along the suckling phase.

Interestingly, at the end of the suckling period, a positive up-regulation of *CLDN1* gene (barrier function) was observed in light piglets, together with an overexpression of some important pro-inflammatory genes (*IL6* and *CXCL2, IDO1*) and stress hormone receptor (*CRHR1, HSD11β1*) compared to heavier piglets. On the one hand, the upregulation of *CLDN1*, an essential structural and functional component of tight junctions (Günzel and Yu, 2013), may suggest an enhancement in the regulation of intestinal permeability. However, the concomitant upregulation of stress hormone signaling (*CRHR1*, *HSD11β1*) and pro-inflammatory genes (*IL6*) and their cofactors (*IDO1*) might be also highlighting the stressful status that light piglets are experiencing. Particularly, *HSD11β1* and *CRHR1* genes have been suggested as stress-induced mediators of an impaired intestinal barrier and hypersecretion on early weaned piglets (Meddings and Swain, 2000; Smith et al., 2010) and in piglets under a lipopolysaccharide challenge (Zhu et al., 2016). The *IDO1* gene encodes the enzyme Indoleamine 2,3-dioxygenase that catalyzes L-tryptophan degradation, but also acts as an immunoregulator which activity seems to be increased under pro-inflammatory circumstances (Frumento et al., 2002).

Taking together these findings, the postnatal growth retardation not only impairs farm economic performance but also negatively affects several physiological functions by increasing pro-inflammatory and stress responses. Among the pathophysiological changes associated with proinflammatory cytokines and stress molecules is the redistribution of nutrients, such as energy and amino acids, that were initially destined for growth (Huntley et al., 2018). It could be speculated that in young pigs, nutrient and immune provisions during the lactation period are not enough to satisfy the needs of the poorly developed piglets that must compete with their littermates for milk provision. In fact, light piglets have shown a deteriorated post-natal and post-weaning development (Bérard et al., 2008; Ji et al., 2017; Rodrigues et al., 2020), with impaired characteristics of organs and carcasses (Rehfeldt and Kuhn, 2006; Bérard et al., 2008) compared to those of piglets with heavier weights. The impaired physiological functions of light piglets observed in the present study support the concept that the unsatisfactory nutrient supply to the fetus/neonate is an important factor influencing their postnatal physiological performance (Larriestra et al., 2006; Farmer and Edwards, 2021).

In conclusion, light BW piglets at birth and at the end of suckling period seem to have an impaired gut development and nutrient absorption, as well as higher pro-inflammatory responses compared to their average weight littermates. Further studies are needed to investigate the short- and long-term consequences of the gut immaturity of light BW piglets, coming from large litters, on their feed efficiency as well as their abilities to cope with post-weaning challenges.

## Abbreviations

ACTB: actin beta
ANPEP: alanyl aminopeptidase membrane
ALPI: alkaline phosphatase intestinal
B2M: beta-2-microglobulin
BW: body weight
CCL: chemokine ligand
CCK: cholecystokinin
CLDN: claudins
CRHR1: corticotropin releasing hormone receptor
DAO: d-amino-acid oxidase
FDR: false discovery rate
FAXDC2: fatty acid hydroxylase domain containing
GCG: glucagon
GPX: glutathione peroxidase
GAPDH: glyceraldehyde-3-phosphate dehydrogenase
GBP1: guanylate binding protein
HSP: heat shock protein
HNMT: histamine N-methyltransferase
HSD11β1: hydroxysteroid (11-beta) dehydrogenase
IDO: indoleamine 2,3-dioxygenase
IGF: insulin-like growth factor
IFNG: interferon gamma
IL: interleukin
IUGR: intrauterine growth-restricted
MT: metallothionein
MUC: mucins
NR: nuclear receptor
OCLN: occludin
PYY: peptide YY
PPARGC1α: peroxisome proliferator activated receptor gamma
REG3G: regenerating family member 3 gamma
SLC: solute carrier
SI: sucrase-isomaltase
SOD: superoxide dismutase
TBP: TATA-box binding protein
TLR: toll-like receptor
TGF-β1: transforming growth factor beta 1
TFF: Trefoil factor
TNF: tumor necrosis factor
ZO: Zonula occludens
